# Recent developments in the detection and management of acute kidney injury

**DOI:** 10.1136/archdischild-2015-309381

**Published:** 2016-08-05

**Authors:** James McCaffrey, Ajaya Kumar Dhakal, David V Milford, Nicholas J A Webb, Rachel Lennon

**Affiliations:** 1 Department of Paediatric Nephrology, Central Manchester University Hospitals NHS Foundation Trust (CMFT), Manchester Academic Health Science Centre (MAHSC), Manchester, UK; 2 KIST Medical College and Teaching Hospital, Imadol, Lalitpur, Nepal; 3 Department of Nephrology, Birmingham Children's Hospital, Birmingham, UK; 4 Wellcome Trust Centre for Cell-Matrix Research, University of Manchester, Manchester, UK

**Keywords:** Acute kidney injury, Biomarker, Stratification

## Abstract

Acute kidney injury (AKI) is a common condition in children admitted to hospital and existing serum and urine biomarkers are insensitive. There have been significant developments in stratifying the risk of AKI in children and also in the identification of new AKI biomarkers. Risk stratification coupled with a panel of AKI biomarkers will improve future detection of AKI, however, paediatric validation studies in mixed patient cohorts are required. The principles of effective management rely on treating the underlying cause and preventing secondary AKI by the appropriate use of fluids and medication. Further therapeutic innovation will depend on improving our understanding of the basic mechanisms underlying AKI in children.

## Introduction

Acute kidney injury (AKI) can be defined as the abrupt loss of kidney function, leading to a decrease in glomerular filtration rate (GFR), and impaired control of acid-base, electrolyte and fluid balance. The term ‘AKI’ has replaced ‘acute renal failure’ as it emphasises that renal dysfunction encompasses a spectrum of disease severity, rather than a single discrete entity. AKI is a common problem in children admitted to hospital, especially among those requiring intensive care, and it is an independent risk factor for increased mortality and severe morbidity.^1^ In addition there are few long-term studies that have assessed the risk of chronic kidney disease (CKD) in children who experience an episode of AKI. In one follow-up study of children admitted to intensive care in Canada, 46.8% were identified as being at risk of CKD between 1 year and 3 years after an episode of AKI.^2^ Here we review the recent progress in identifying early AKI biomarkers to improve detection; we describe key aspects of managing AKI in children and we highlight areas for future development.

### Creatinine as an AKI biomarker

**Figure 1:**
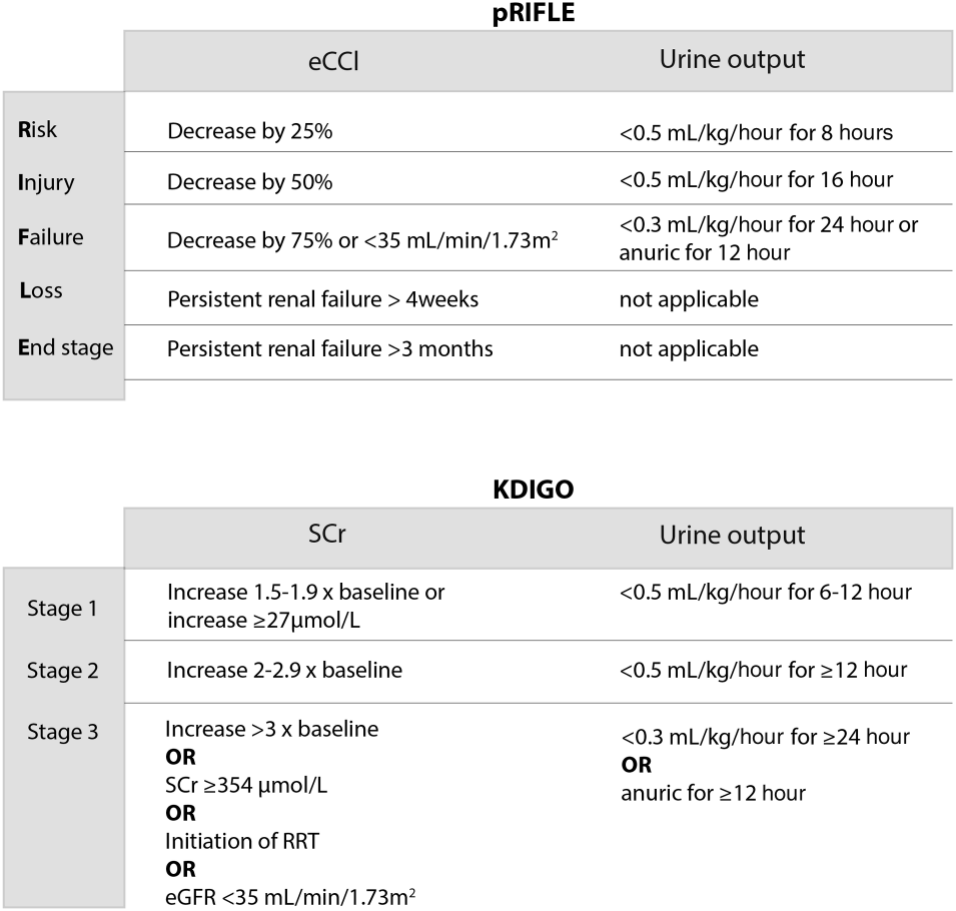
Diagnostic criteria for AKI. Both pRIFLE and KDIGO classification systems stratify AKI into levels of severity, determined by changes in serum creatinine (SCr)/estimated creatinine clearance (eCCl) and changes in urine output. AKI severity strata can be reached through fulfilling criteria for either changes in SCr/eCCl or changes in urine output. AKI, acute kidney injury; pRIFLE, paediatric Risk or renal dysfunction, Injury to the kidney, Failure of kidney function, Loss of kidney function, and End-stage renal disease. KDIGO, Kidney Disease: Improving Global Outcomes; RRT, renal replacement therapy; eGFR, estimated glomerular filtration rate.

The diagnosis of AKI has traditionally relied on measurements of serum creatinine (SCr) as a marker of GFR and/or monitoring of urine output. However, SCr is a poor biomarker for AKI and there is no consensus AKI definition, as illustrated by the existence of more than 30 definitions in the published literature.^3^ Two of the most widely used definitions for paediatric AKI are the paediatric Risk or renal dysfunction, Injury to the kidney, Failure of kidney function, Loss of kidney function, and End-stage renal disease (pRIFLE) and Kidney Disease Improving Global Outcomes (KDIGO) classifications (figure 1). The use of muscle-derived creatinine as a biomarker for AKI is based on the observation that it is freely filtered by the healthy glomerulus, but creatinine filtration becomes less efficient as renal damage increases in severity, and SCr levels rise as a consequence. However, ‘normal’ baseline SCr varies widely due to many interindividual variables including muscle mass, age and sex.^4^ SCr is also a late and insensitive marker of renal damage; levels only rise significantly once 25–50% of renal function has been lost^5^ and there is a temporal dissociation between reduced GFR and increased SCr.^6^ Furthermore, relatively small changes in SCr levels may reflect significant pathology: in a study involving children with acute decompensated heart failure, an inhospital SCr increase of >27 µmol/L was independently associated with increased patient mortality, and need for mechanical ventilation.^7^ Other limitations of SCr measurements include a poor correlation with GFR outside of steady-state filtration (GFR changes rapidly during AKI), and the dissociation between renal function and SCr in patients receiving dialysis (dialysis removes creatinine). Additionally, in diseases such as lupus nephritis, significant kidney injury can coexist with preserved GFR and maintenance of a normal SCr.^8^ The lack of consensus regarding the definition of AKI is problematic, as it has led to a wide variation in reported epidemiological, and morbidity and mortality data. As SCr-based methods of monitoring renal function often hamper timely diagnosis of AKI, this may also partially explain the repeated failure of novel therapeutic interventions for AKI, as significant disease progression has already occurred by the time the trial therapy is administered.

## AKI epidemiology

The largest epidemiological study of paediatric AKI to date identified 2 644 263 hospital admissions (encompassing both general and critical care populations) in hospitals throughout the USA, and reported an AKI incidence of 3.9 cases per 1000 admissions.^9^ Children experiencing AKI were identified using International Classification of Disease, Ninth Revision, Clinical Modification (ICD-9-CM) codes. AKI incidence increased with age, and was greatest in patients aged 15–18 years (6.6 events per 1000 admissions). Mortality within the AKI cohort was significantly higher than among non-AKI admissions (15.3% vs 0.6%). A further study from a single tertiary referral centre in southern Thailand reported an incidence of AKI (defined by a sudden increase in SCr of >177 µmol/L or a doubling of SCr levels) of 4.6–9.9 cases per 1000 admissions between 1995 and 2004, with an overall mortality of 41.5%.^10^ Children requiring admission to a paediatric intensive care unit (PICU) are at much higher risk of developing AKI: in a US study involving 150 critically ill children requiring invasive mechanical ventilation, 82% developed AKI by pRIFLE criteria (48.8% of these reached pRIFLE R, 26.0% reached pRIFLE I and 25.2% reached pRIFLE F).^3^ The aetiology of AKI has also changed recently from primary renal disease to renal involvement secondary to systemic insults such as sepsis and iatrogenic interventions such as the use of medication with nephrotoxic potential.^11^

## Advances in AKI detection

There is a clear need to identify more sensitive and earlier biomarkers of AKI. Perhaps the best example of a biomarker that can reliably identify the abrupt onset of organ dysfunction is the use of troponin I for the diagnosis of acute myocardial infarction (AMI) in the adult population. However, the search for an analogous AKI biomarker is hindered by the difficulties in identifying patients at risk of renal dysfunction. In AMI, patients typically present with acute chest pain, which alerts the clinician to the possibility of a coronary event and triggers biomarker evaluation using troponin I. As with any biomarker, the predictive performance dramatically increases if evaluation only involves patients already assessed as ‘at-risk’ of a particular condition.^12^ For example, when troponin I is measured in unselected critically ill patients, elevated troponin I is not indicative of AMI.^13^ In AKI, there is no obvious equivalent of ‘chest pain’ to aid the clinician in prebiomarker clinical risk stratification. This is problematic because children with AKI are often identified after the window of opportunity for potentially successful intervention has passed. Thus, recent developments in AKI detection may be divided into: (1) determining patients who are risk of AKI; and (2) discovery of early and sensitive biomarkers for the assessment of patients deemed at risk of AKI.

### AKI risk stratification

**Figure 2:**
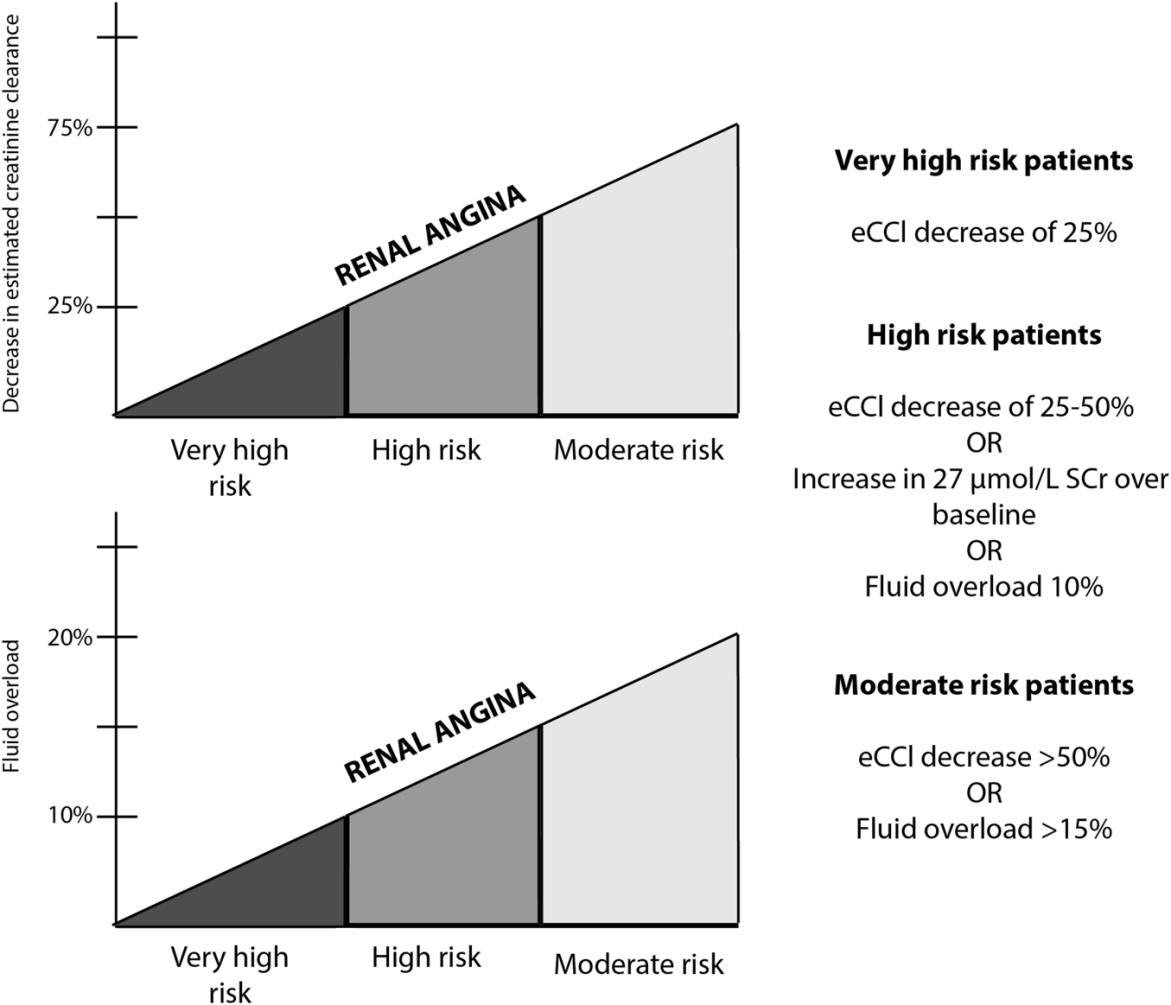
Renal angina threshold to identify children who may require evaluation with acute kidney injury (AKI) biomarkers. The graphs depict risk of AKI versus decrease in estimated creatinine clearance (eCCl) and percentage increase in fluid overload. There are three risk groups defined for the paediatric intensive care unit (PICU) population: very high risk (intubated plus the presence of at least one vasopressor or inotrope), high risk (history of solid organ or bone marrow transplant) and moderate risk (PICU admission). Less evidence of injury is required for higher risk groups to fulfil ‘renal angina’ criteria and further validation with AKI biomarkers is required. (Adapted from Basu *et al*^17^).

**Figure 3:**
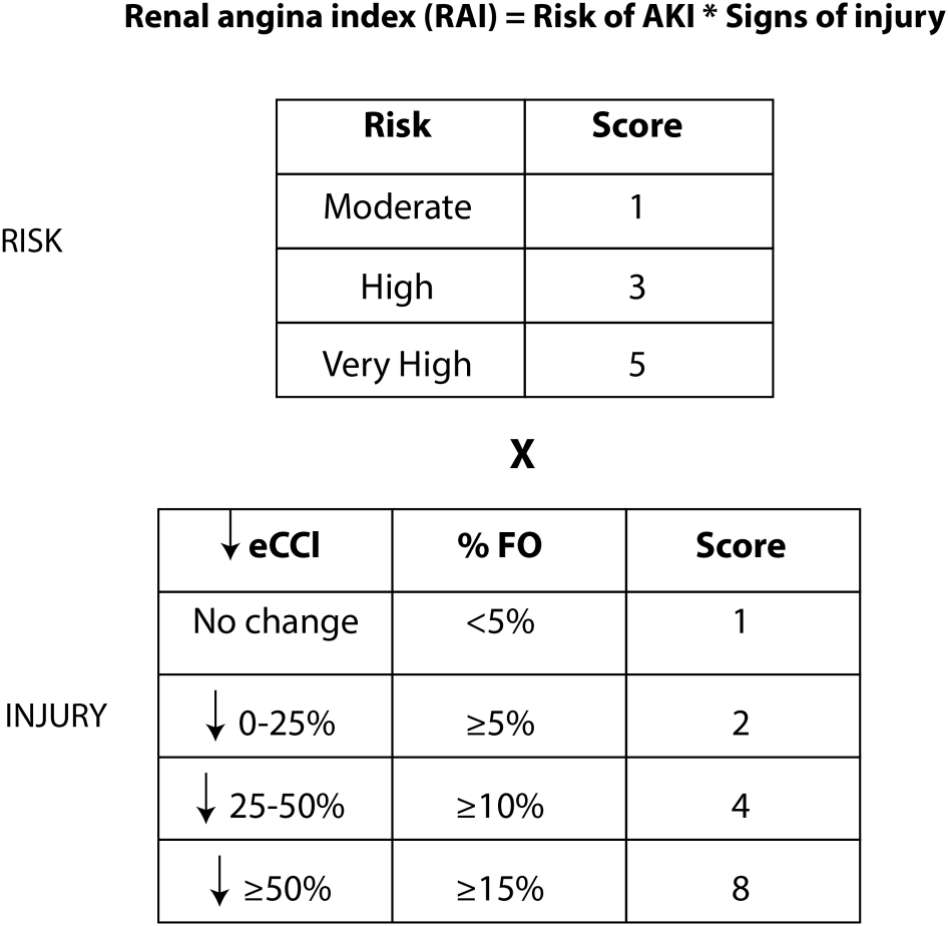
The renal angina index (RAI). Patients are stratified depending on their ‘risk’ of developing acute kidney injury (AKI) and their signs of injury. There are three risk groups: moderate (paediatric intensive care admission), high (prior stem cell transplantation) and very high (ventilation and requirement for either an inotrope or vasopressor). The worse parameter between change in estimated creatinine clearance (eCCl) from baseline and percentage fluid overload (% FO) yields an injury score. The resultant RAI can range from 1 to 40. A cut-off value of ≥8 is used to determine fulfilment for ‘renal angina.’ (Adapted from Basu *et al*^17^).

Although any hospitalised child may develop AKI, studies attempting to risk stratify paediatric patients have focused on those admitted to PICU. The search for AKI biomarkers to use in patients deemed to be at risk of developing AKI is viewed by many researchers as closely analogous to the identification of troponin I for use in AMI. Indeed Goldstein *et al*,^12^ have proposed a definition of ‘renal angina’ to initiate assessment of ‘renal troponin I’ AKI biomarker(s). This proposal draws on data demonstrating that a doubling of SCr (pRIFLE I) in children requiring intensive care was associated with 27.4% mortality compared with an overall PICU mortality of 2.4%^14^ and the aforementioned study identifying small increases in SCr of >27 µmol/L as an independent predictor of patient mortality in children with acute decompensated heart failure.^7^ Additionally, increasing degrees of relative fluid accumulation or percentage of fluid overload at the time of initiating renal replacement therapy (RRT) in children with AKI is independently associated with mortality.^15^ Children who have received stem cell transplants are particularly vulnerable to the deleterious effects of fluid accumulation.^16^ The empirical clinical model of ‘renal angina’ for the prediction of subsequent AKI developed by Goldstein *et al*, identifies three risk groups: very high risk (intubated plus the administration of at least one vasopressor or inotrope), high risk (history of solid organ or bone marrow transplant) and moderate risk (PICU admission). Less severe signs of injury (SCr change or percentage fluid overload) are required for higher risk groups to fulfil the ‘renal angina’ threshold (figure 2). Subsequently, a quantifiable ‘renal angina index’ was developed which is a composite of patient AKI risk and early signs of injury. The index score can range from 1 to 40, with a cut-off of ≥8 used to fulfil the criteria for ‘renal angina’ (figure 3).^17^ As an alternative, some have proposed that the fulfilment of pRIFLE R criteria should be the definition of renal angina.^12^

### AKI biomarkers

Timely diagnosis of AKI requires the measurement of an early and specific biomarker(s) in patients deemed at risk of developing AKI. Table 1 shows selected studies of biomarkers in paediatric AKI. As described above, SCr is a poor AKI biomarker but recent research efforts have identified several promising novel AKI biomarkers including: neutrophil gelatinase-associated lipocalin (NGAL), kidney injury molecule 1 (KIM-1), interleukin 18 (IL-18), liver-type fatty acid-binding protein (L-FABP), neutrophil elastase-2 (Ela-2) and cystatin C (Cys-C). NGAL is a widely expressed protein of the lipocalin family, whose main physiological role is as a bacteriostatic agent in the innate immune response. Filtered and secreted NGAL is released into the urine, and there is also megalin-dependent tubular reabsorption into plasma.^24^ NGAL production is dramatically upregulated following renal injury. KIM-1 is a transmembrane protein that promotes the phagocytosis of apoptotic bodies and necrotic debris. Basal expression of KIM-1 is low in the normal kidney, but production in proximal tubule cells is upregulated following renal injury, leading to increased urinary concentration following renal injury. IL-18 is a proinflammatory cytokine that promotes acute injury by inducing the upregulation of inflammatory meditators such as tumour necrosis factor α via the nuclear factor-κB pathway. L-FABP is predominantly localised in the proximal tubule and is a renoprotective protein, which promotes the metabolism of long-chain and very long-chain fatty acids, as well as having antioxidant properties. Ela-2 is a serine protease secreted by neutrophils in response to bacterial infection and inflammation. The majority of the novel AKI biomarkers are early markers of injury because their expression is increased following renal *structural* damage. This is in contrast to SCr, which is a *functional* marker of renal damage. Another functional marker of AKI is Cys-C, which is a protease inhibitor produced at a constant rate by most nucleated cell types, whose elimination is almost exclusively via glomerular filtration. Although serum Cys-C levels rise within a similar time frame to SCr (and do not therefore provide earlier warning of AKI), Cys-C has a practical advantage over SCr in that it reflects the GFR independently of body height and muscle mass. The reference range of Cys-C varies little after the 1st year of life, thus avoiding the need to calculate an estimated GFR.^25^ No study has investigated all of the described biomarkers in a single patient cohort, but we recently reported changes in Cys-C, plasma NGAL, urinary NGAL and KIM-1 rise by comparison to changes in SCr prior to AKI.^22^

**Table 1:** Studies of new biomarkers for AKI in children

Study	Biomarkers	Comments
Du *et al*^18^	Urine KIM-1, NGAL, β2M, IL-18, osteopontin	232 children presenting to emergency care. KIM-1, NGAL and β2M all demonstrated good accuracy with 25%–50% reduction in eCCl.
Zappitelli *et al*^19^	Plasma: Cys-C	288 children undergoing cardiac surgery. Postoperative Cys-C predicted length of stay in PICU.
Buelow *et al*^20^	Urine: NGAL, IL-18	20 children undergoing cardiac surgery. NGAL and IL-18 early predictive biomarkers of AKI.
Genc *et al*^21^	Urine: KIM-1	48 premature babies. Serial urinary KIM-1 was a maker of kidney injury.
Basu *et al*^17^	Plasma: NGAL, MMP-8, Ela-2	214 children admitted to PICU with sepsis. Biomarker performance improved in combination with risk stratification.
McCaffrey *et al*^22^	Plasma: NGAL, Cys-C Urine: NGAL, KIM-1	Mixed cohort of 49 children in PICU. Plasma NGAL predicted AKI; Cys-C mirrored change in SCr.
Westhoff *et al*^23^	Urine: TIMP-2 and IGFBP-7	133 mixed cohort of children. The [TIMP-2]•[IGFBP-7] product diagnosed AKI and predicted adverse outcomes.

AKI, acute kidney injury; B2M, Beta-2 microglobulin; Cys-C, cystatin C; eCCl, estimated creatinine clearance; IGFBP, insulin-like growth factor binding protein; IL, interleukin; KIM, kidney injury molecule; MMP, matrix metalloproteinase; NGAL, neutrophil gelatinase-associated lipocalin; PICU, paediatric intensive care unit; SCr, serum creatinine; TIMP, tissue inhibitor of metalloproteinase.

Most AKI biomarker studies in the paediatric population reported to date have focused on very specific patient groups. For example, in the paediatric cardiopulmonary bypass surgery population, NGAL (in plasma and urine) is the best performing single AKI biomarker.^26^ These patients display a large degree of demographic homogeneity, and a known onset and duration of ischaemic injury. In contrast, AKI biomarker performance is much more variable when tested in more ‘real-life’ cohorts of hospitalised children, who display more heterogeneity in their demographics and AKI aetiology. Biomarker performance is usually quantified using receiver operator characteristic curves to calculate the area under the curves (AUCs). An AUC of 0.5 means a biomarker is performing no better than if the clinician were assessing whether a patient had AKI or not by flipping a coin, whereas a value of 1.0 signifies a perfect biomarker. An AUC of 0.75 is generally considered to be a good biomarker. Du *et al*,^18^ conducted a prospective study involving 252 children presenting to a paediatric emergency care centre (ECC) in the USA, and found AUCs to predict AKI of pRIFLE I severity as follows: NGAL (0.83), KIM-1 (0.77) and IL-18 (0.68), suggesting these urinary biomarkers perform well to identify which patients have AKI on presentation to a paediatric ECC. The authors also identified a ‘real-world’ problem when attempting to allocate the patients into pRIFLE strata, as perhaps not surprisingly only 27% of patients had a baseline SCr recorded within the 3 months prior to the ECC visit. In the absence of a recorded individual baseline SCr, presumed estimated creatinine clearances of 120 mL/min/1.73 m^2^ are typically used. The lack of an individual baseline SCr may be problematic: in our own study investigating a paediatric PICU population, only 8.2% of patients had preadmission SCr values, and 75% of patients with a recorded prior SCr value were above the presumed baseline of 120 mL/min/1.73 m^2^.^22^ This suggests that baseline renal function may be underestimated for a proportion of children when individual baseline SCr values are unavailable, leading to underdiagnosis of AKI using pRIFLE criteria.

Data demonstrating that the combined use of risk stratification and the use of new biomarkers improves detection of AKI come from a study involving 214 children admitted to PICU with sepsis.^17^ The AUC for severe AKI (defined by KDIGO ≥stage 2) on day 3 of admission for the renal angina index alone was 0.80. Plasma NGAL (AUC 0.72) and Ela-2 (AUC 0.72) individually demonstrated marginal discrimination for severe AKI. Interestingly, the combination of the renal angina index with plasma NGAL and Ela-2 showed excellent predictive power for subsequent AKI (AUC 0.88). Biomarker positivity in patients without ‘renal angina’ did not predict day 3 AKI, underlining the need for pretest risk stratification. Data from adult patients also suggest a combination of biomarkers may outperform any individual AKI biomarker alone. Kashani *et al* performed a multicentre study in critically ill adults and found that urinary insulin-like growth factor binding protein 7 (IGFBP-7) and tissue inhibitor of metalloproteinase (TIMP)-2 (both are inducers of G_1_ cell cycle arrest) were the best performing biomarkers for AKI from an extensive list of 340 candidate biomarkers (including KIM-1, NGAL, IL-18, Cys-C and L-FABP). The AUCs for IGFBP-7 and TIMP-2 were 0.76 and 0.79, respectively, while no other biomarker achieved an AUC >0.72. The combination of TIMP-2 and IGFBP-7 increased the AUC to 0.80.^27^ On the basis of these data, the US food and drug administration has permitted marketing of NephroCheck for adults. This immunoassay measuring TIMP-2 and IGFBP-7^28^ is the first device to use biomarkers of kidney damage to detect early AKI and it represents a major advance in the detection of AKI for adults. Data regarding its performance in everyday clinical use are eagerly awaited. The use of TIMP-2 and IGFBP-7 in the paediatric population has not been extensively investigated, but one small study involving a heterogeneous population of children and neonates found NephroCheck had a moderate performance in predicting the need for RRT (AUC 0.67).^23^ Further paediatric data are required to assess these and other combinations of AKI biomarkers.

## Management

There are aspects of our current management of children with, or at risk of developing, AKI that could be more widely implemented to improve outcomes. This includes the timely measurement of SCr and recognition of changes in SCr. To this end the National Health Service in England recently mandated the introduction of electronic alerts for adults and children with AKI. The alerts are based on changes in SCr where AKI 1 is baseline SCr ×1.5; AKI 2 is baseline SCr ×2 and AKI 3 is 3× baseline SCr/upper limit of the SCr reference interval.^29^ Although a recent adult study did not show a benefit of the alerts system alone,^30^ it is anticipated that combining AKI alerts with an AKI pathway for intervention will improve outcomes. Indeed the early diagnosis and management of AKI decreases mortality and long-term morbidity and appropriate early consultation with nephrology has been shown to improve renal survival and long-term outcome.^31^ Another requirement in the management of AKI is the identification of a precipitating factor. This necessitates a thorough history, examination and laboratory investigation to identify the underlying cause. Whether there is associated hypovolaemia, sepsis or postrenal obstruction, directed management should be instituted to treat the underlying cause of AKI. It is also essential that children have appropriate monitoring during an evolving illness and systems such as the Paediatric Early Warning System enable the identification of deteriorating clinical signs.^32^ For children with, or at high risk of, AKI, the monitoring of urine output and regular measurements of SCr and electrolytes should be instituted early to detect the AKI. Similarly ultrasound of the urinary tract is an important investigation to exclude postrenal obstruction and ideally should be performed within 24 hours of hospital admission.^33^

Evidence to support drug treatment for AKI is limited. While it is commonplace to use a trial of loop diuretics in children with fluid overload, the use of diuretics in the patients with hypovolaemia is associated with poor outcomes and the available evidence does not support the routine use of diuretics to prevent AKI.^33^ There is also controversy regarding the role of low-dose dopamine and routine use of dopamine in patients with AKI should be avoided since it is thought to worsen renal perfusion.^34^ Trials of new treatments for AKI have been disappointing to date and few of these have included children. Of 126 registered AKI clinical trials in 2011, 118 were in adults and only 8 were in children. Of these trials 65% were prevention studies, investigating a timed insult (eg, cardiac surgery). In addition many of these trials were inadequately powered; few were using agreed AKI definitions and 21/22 treatment trials were based in the intensive care unit and were investigating RRT.^35^ As such there is an urgent need to explore novel therapeutic agents and to revisit existing therapies in order to review potential roles in the management of AKI.

Preventing episodes of secondary AKI can also reduce the overall incidence of AKI. Secondary injury can be caused by inappropriate doses of medication with nephrotoxic potential. Examples of these agents include aminoglycosides, non-steroidal anti-inflammatory drugs, calcineurin inhibitors and inhibitors of the renin-angiotensin-aldosterone pathway. These agents should be avoided if children are dehydrated and therefore at risk of AKI. Indeed patients receiving these treatments regularly should be issued with ‘sick day rules’ which provide guidance about the circumstances in which to omit potentially nephrotoxic medicines.^36^ Appropriate fluid management is also critical and the restoration of adequate blood volume is a priority in the early management of AKI.^37^ Overzealous administration of fluid causing fluid overload however, increases the mortality. The appropriate choice of fluid for different causes and stages of AKI is yet not known. Hyperchloraemic metabolic acidosis can be caused by 0.9% saline and in a sequential period pilot study, 0.9% saline was also found to increase risk of AKI in critically ill adults by comparison to chloride-restricted intravenous fluids.^38^ However a more recent and larger trial found no difference between 0.9% saline and chloride-restricted fluids in terms of the incidence of AKI, the rate of RRT and mortality in adult patients.^39^

With established AKI or resistant fluid overload, RRT is required and here continuous haemofiltration or peritoneal dialysis are more suitable for haemodynamically unstable patients as opposed to intermittent haemodialysis, which is associated with more hypotensive episodes.^40^ Importantly, nutrition should not be overlooked in children with AKI as underfeeding can be accentuated by AKI.^41^ The enteral mode of nutrition is preferred and this should start in the early phase of AKI, unless there are contraindications such as active colitis. Finally it is important that children with AKI have appropriate follow-up to ensure recovery, prevent further episodes of AKI and to screen for progression to CKD. Since progression of kidney disease is silent, there is a strong argument for lifelong screening of blood pressure and urinalysis in children who have experienced an episode of AKI.

## Summary

AKI is a common condition in children admitted to hospital and existing serum and urine biomarkers are insensitive. There have been significant developments in stratifying the risk of AKI in children and also in the identification of new AKI biomarkers. Risk stratification coupled with a panel of AKI biomarkers will improve future detection of AKI, however paediatric validation studies in mixed patient cohorts are required. The principles of effective management rely on treating the underlying cause and preventing secondary AKI by the appropriate use of fluids and medication. Further therapeutic innovation will depend on improving our understanding of the basic mechanisms underlying AKI in children.
